# Estimating Physical Activity and Sedentary Behavior in a Free-Living Context: A Pragmatic Comparison of Consumer-Based Activity Trackers and ActiGraph Accelerometry

**DOI:** 10.2196/jmir.5531

**Published:** 2016-09-07

**Authors:** Sjaan R Gomersall, Norman Ng, Nicola W Burton, Toby G Pavey, Nicholas D Gilson, Wendy J Brown

**Affiliations:** ^1^ Centre for Research on Exercise, Physical Activity and Health School of Human Movement and Nutrition Sciences The University of Queensland Brisbane Australia

**Keywords:** activity tracker, physical activity, sedentary behavior, accelerometry, Fitbit, Jawbone

## Abstract

**Background:**

Activity trackers are increasingly popular with both consumers and researchers for monitoring activity and for promoting positive behavior change. However, there is a lack of research investigating the performance of these devices in free-living contexts, for which findings are likely to vary from studies conducted in well-controlled laboratory settings.

**Objective:**

The aim was to compare Fitbit One and Jawbone UP estimates of steps, moderate-to-vigorous physical activity (MVPA), and sedentary behavior with data from the ActiGraph GT3X+ accelerometer in a free-living context.

**Methods:**

Thirty-two participants were recruited using convenience sampling; 29 provided valid data for this study (female: 90%, 26/29; age: mean 39.6, SD 11.0 years). On two occasions for 7 days each, participants wore an ActiGraph GT3X+ accelerometer on their right hip and either a hip-worn Fitbit One (n=14) or wrist-worn Jawbone UP (n=15) activity tracker. Daily estimates of steps and very active minutes were derived from the Fitbit One (n=135 days) and steps, active time, and longest idle time from the Jawbone UP (n=154 days). Daily estimates of steps, MVPA, and longest sedentary bout were derived from the corresponding days of ActiGraph data. Correlation coefficients and Bland-Altman plots with examination of systematic bias were used to assess convergent validity and agreement between the devices and the ActiGraph. Cohen’s kappa was used to assess the agreement between each device and the ActiGraph for classification of active versus inactive (≥10,000 steps per day and ≥30 min/day of MVPA) comparable with public health guidelines.

**Results:**

Correlations with ActiGraph estimates of steps and MVPA ranged between .72 and .90 for Fitbit One and .56 and .75 for Jawbone UP. Compared with ActiGraph estimates, both devices overestimated daily steps by 8% (Fitbit One) and 14% (Jawbone UP). However, mean differences were larger for daily MVPA (Fitbit One: underestimated by 46%; Jawbone UP: overestimated by 50%). There was systematic bias across all outcomes for both devices. Correlations with ActiGraph data for longest idle time (Jawbone UP) ranged from .08 to .19. Agreement for classifying days as active or inactive using the ≥10,000 steps/day criterion was substantial (Fitbit One: κ=.68; Jawbone UP: κ=.52) and slight-fair using the criterion of ≥30 min/day of MVPA (Fitbit One: κ=.40; Jawbone UP: κ=.14).

**Conclusions:**

There was moderate-strong agreement between the ActiGraph and both Fitbit One and Jawbone UP for the estimation of daily steps. However, due to modest accuracy and systematic bias, they are better suited for consumer-based self-monitoring (eg, for the public consumer or in behavior change interventions) rather than to evaluate research outcomes. The outcomes that relate to health-enhancing MVPA (eg, “very active minutes” for Fitbit One or “active time” for Jawbone UP) and sedentary behavior (“idle time” for Jawbone UP) should be used with caution by consumers and researchers alike.

## Introduction

Regularly participating in physical activity and minimizing time spent in sedentary behavior are associated with a significantly reduced risk of poor health outcomes, including cardiovascular disease, overweight and obesity, and all-cause mortality [1,2]. Despite the health benefits, many individuals are physically inactive and spend large amounts of time sedentary [3,4]; Australians spend 50% to 70% of their waking time being sedentary [5] and almost 60% of Australian adults are classified as insufficiently active (<150 minutes/week of moderate-to-vigorous physical activity) [3]. Strategies for increasing physical activity and reducing time spent sedentary are important to reverse these trends and for preventing poor health outcomes.

Activity trackers are becoming increasingly popular for monitoring physical activity and sedentary behavior, and for promoting positive behavior change [6]. Activity trackers are commonly waist- or wrist-worn devices that include a range of sensors for self-monitoring behavior. These devices typically sync with a Web- or app-based interface, which provides summary data and individual feedback on behaviors. Common types of activity data from these devices include number of steps, time spent in physical activity by intensity, and time spent “idle.” The devices also have additional functions that can be used to support behavior change, such as goal setting (eg, 10,000 steps per day), prompts/cues, and social networking and accountability [7]. The uptake of these devices, both in the consumer market and in research, has been rapid [8-10]. It is estimated that more than 50 million smartwatch and health and fitness trackers were sold worldwide in 2015 and it is predicted that this figure will reach more than 80 million in 2016 [11].

Given the commercial availability of next-generation activity trackers and the rapid rate of uptake by consumers and researchers, it is important to understand their accuracy. Previous studies investigating the Fitbit One (worn on the hip) or the wrist-worn Jawbone UP in laboratory settings have demonstrated a high level of accuracy for physical activity outcomes compared with reference methods, with relative differences and correlations ranging from approximately 2% to 20% and ≥.97, respectively [12-15]. However, given their controlled settings, these studies have limited ecological validity. Only two studies have investigated the validity of a Fitbit or Jawbone device in free-living settings [16,17]. Although these studies have also demonstrated acceptable validity, correlations were lower than in controlled settings, ranging from .8 to .9 [16,17]. In addition, no previous studies have investigated the sedentary behavior features of the Jawbone UP (eg, idle time) [18]. Further research is required in free-living conditions, which reflect how these devices are used day to day.

The aim of this study was to compare Fitbit One and Jawbone UP estimates of steps, moderate-to-vigorous physical activity (MVPA), and sedentary behavior with data from the ActiGraph GT3X+ accelerometer in a free-living context.

## Methods

Data were collected as part of a larger, 12-week physical activity intervention study that included three groups that were randomly allocated to wear a Fitbit One, Jawbone UP, or standard pedometer. The aim of this larger study was to compare the efficacy of the three devices to increase physical activity. Outcomes were measured at baseline, mid-, and post-intervention in August, September, and October 2014 using the ActiGraph GT3X+ accelerometer. Data for this substudy were collected at mid- and post-intervention when participants concurrently wore an ActiGraph GT3X+ accelerometer. Demographic and anthropometric data for this study were collected at baseline. Ethical approval for this study was obtained from The University of Queensland Ethics Committee (#2014000766).

### Recruitment and Participants

Participants were recruited in July 2014 via convenience sampling at three campuses of a large Australian metropolitan university via an email advertisement to staff that included study information and participant eligibility criteria. The target sample size was 15 participants per group based on sample size calculations for the intervention trial. People indicated interest via return email and were then screened for eligibility via telephone interview. To be eligible, participants had to be healthy, ambulatory, aged between 18 and 65 years, have accumulated less than 150 minutes of MVPA in the past week (assessed using the Active Australia Survey [19,20]), and own or have access to a mobile phone compatible with both the Fitbit One and Jawbone UP. People who met all eligibility criteria were then invited to an individual face-to-face appointment where they provided written informed consent prior to data collection. At the conclusion of the study, participants received an AU $50 gift card gratuity.

## Measures

### Fitbit One

**Figure 1:**
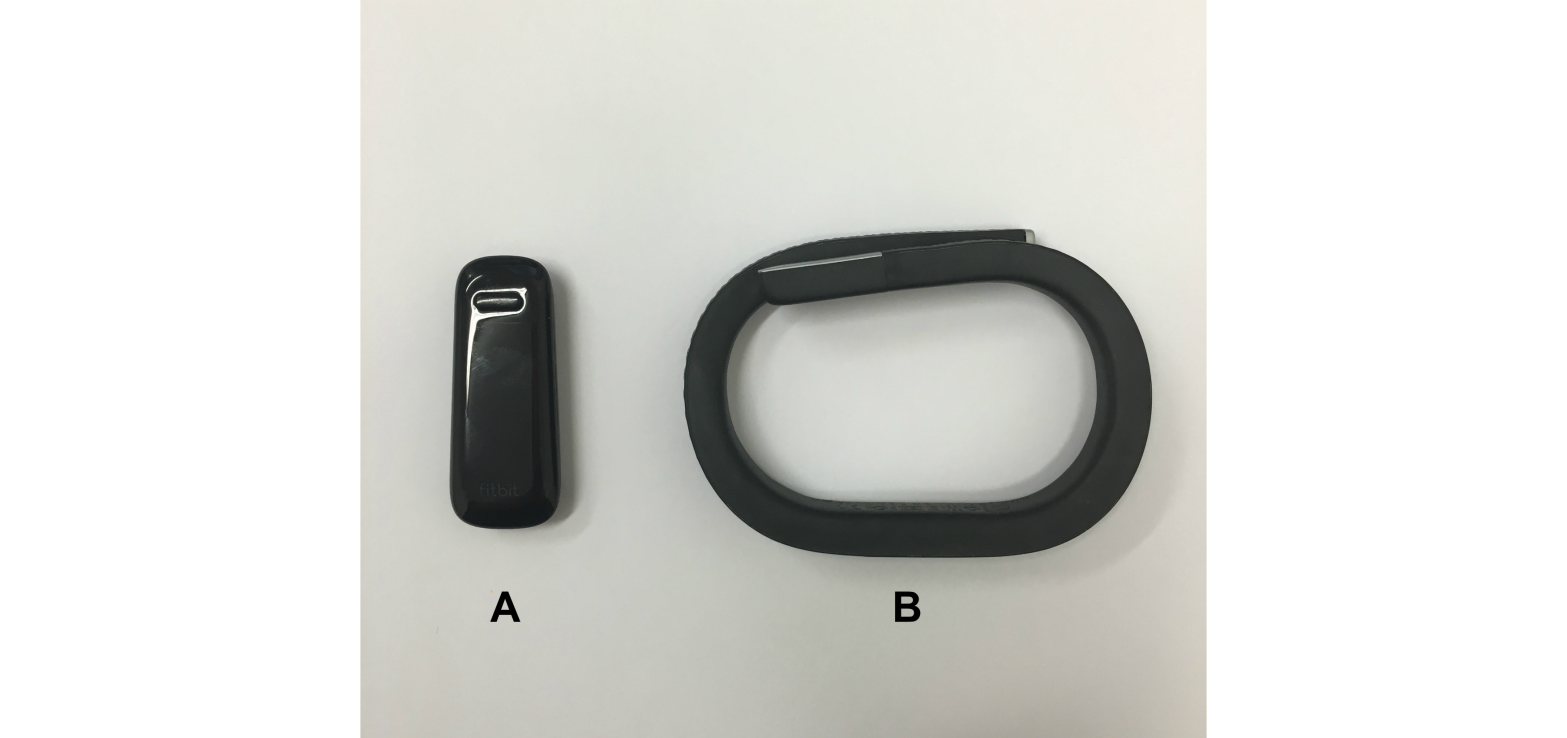
Fitbit One (A) and Jawbone UP (B).

The Fitbit One (Fitbit Inc, San Francisco, CA, USA) is a small (48.0 × 19.3 × 9.65 mm), light-weight (8 g), consumer-based activity tracker that includes a three-axis accelerometer and an altimeter (Figure 1). Outcomes from this device include steps, floors climbed, distance traveled, calories burned, and active minutes. At the time these data were collected, the active time variable was labeled “very active minutes.” This was not well defined by Fitbit Inc; the explanation was limited to time spent in “higher intensity” exercise [21]. For this study, *very active minutes* was defined as time spent in MVPA. The Fitbit One syncs via Bluetooth Low Energy to a mobile device or a computer. Data can be viewed using the Web-based platform or the Fitbit mobile phone app, and immediate feedback on cumulative data for the current day is provided on the organic light-emitting diode (OLED) display. The device has a battery life of 10 to 14 days and stores 7 days of detailed, minute-by-minute data as well as daily summary data for the preceding 23 days. The device is not waterproof and the manufacturer’s instructions indicate that the device can be worn on a belt, bra, or in a pocket.

### Jawbone UP

The Jawbone UP (Jawbone, San Francisco, CA, USA) is a light (19-23 g), wrist-worn, consumer-based activity tracker that includes a three-axis accelerometer (Figure 1). Outcomes from this device include steps, distance traveled, active time, calories burned, and longest idle time. No manufacturer definition for “active time” or “longest idle time” could be identified. For this study, *active time* was defined as time spent in MVPA and *longest idle time* was defined as longest sedentary bout. The Jawbone UP syncs via the 3.5 mm headphone jack and is only compatible with mobile devices; therefore, data are viewed using the associated mobile phone app and there is no display on the device for immediate feedback. The wrist-worn activity tracker comes in three sizes (small: 14.0 × 15.5 cm; medium: 15.5 × 18.0 cm; large: 18.0 × 23.0 cm), has a battery life of approximately 10 days, can store up to 9 months of data, and is not waterproof. The manufacturer’s instructions indicate that the device can be worn on either the dominant or nondominant wrist, with the wear location reported in the Settings. For this study, participants could choose which wrist they wanted to wear the device on and were encouraged to ensure that the correct location was entered in the Settings.

For both trackers, daily estimates for the outcomes of interest (Fitbit One steps and active minutes; Jawbone UP active time and longest idle time) were extracted from the users’ accounts and entered into an Excel spreadsheet by a research assistant. Data were included for days that there was a corresponding valid day of accelerometry data. Participants were instructed to wear the devices during waking hours, removing them for water-based activities or contact sports, but were not required to keep wear logs in order to improve the free-living fidelity of the devices over the 12-week intervention. Participants were able to input activity sessions, such as swimming/contact sports, through the “log workout” function in the Jawbone UP app and the “track exercise” feature in the Fitbit app.

### Accelerometry

The comparison instrument for this study was the ActiGraph GT3X+ accelerometer (Pensacola, FL, USA). Participants were asked to wear the accelerometer on their right hip for 7 days at each measurement occasion, except when sleeping, during water-based activities, or engaging in contact sport. Participants were also asked to complete a brief log to record and monitor on/off times, wake and sleep times, and the duration and reason if the monitor was removed for more than 10 minutes. The ActiGraph GT3X+ accelerometer was initialized with a 30 Hz sampling frequency and raw data from .gt3x files were converted to 30-second epoch data files prior to analysis. A *valid day* was defined as a minimum wear time of 10 hours/day, with *non-wear time* defined as 60 minutes or more of consecutive activity counts of zero, with a spike tolerance of 2 minutes and 100 counts/minute [22,23]. For all valid days, daily estimates of steps (steps/day), time spent in MVPA (minutes/day), and longest sedentary bout (minutes/day) were derived from the vertical axis data using ActiLife software version 6 (ActiGraph, Pensacola, FL, USA) using cutpoints of less than 100 counts/minute for sedentary [24] and more than or equal to 2020 counts/minute for moderate-to-vigorous intensity activity [22].

The ActiGraph GT3X+ accelerometer has been shown to have good reliability (intraclass correlation coefficient [ICC]=.97 when tested using a motorized vibration table) [25]. Few studies have been published on the validity of the GT3X+ version of the ActiGraph accelerometer specifically; however, previous versions of the ActiGraph accelerometer (CSA and GT1M) have good waist-worn validity in treadmill walking and running compared with indirect calorimetry (*r*=.56, *P*<.001 and *r*=.53, *P*<.05, respectively) in adults [26,27]. A recent study has demonstrated acceptable agreement between steps estimated by the ActiGraph GT3X+ at moderate-high walking speeds in a laboratory setting (ICC .72-.99 compared with direct observation) and in free-living situations (ICC=.90; compared with Yamax Digiwalker) [28].

### Demographic Variables and Anthropometry

Written questionnaire items were used to collect information on gender, date of birth, and level of education. Standing height and weight were measured using a stadiometer (217 stadiometer, SECA, Hamburg, Germany) and an electronic scale (Sensa 804, SECA, Hamburg, Germany) according to protocols developed by the International Society for the Advancement of Kinanthropometry [29]. Each variable was measured twice and the mean obtained. If the first and second measures varied by more than 10%, a third was measured and the median of the three values was recorded. The same equipment was used for all participants. Body mass index (BMI) was determined as weight (kg)/height^2^ (m).

### Statistical Analyses

Descriptive statistics (n, mean, standard deviation, and prevalence) were calculated for demographic and physical measures. Absolute agreement was examined using ICCs and 95% confidence intervals. Correlation was assessed using Pearson correlation coefficient (*r*) or Spearman rank correlation coefficient (ρ) when data were non-normally distributed with 95% confidence intervals. The strength of correlation coefficients was interpreted based on the following definitions: weak (*r*=.5), moderate (*r*=.5-.7), and strong (*r* ≥.7). Bland-Altman plots [30] were used to examine the differences between all outcomes, with mean bias and 95% limits of agreement reported. After visual examination of the plots, linear regression was used to examine whether mean difference and limits of agreement varied across mean values of Fitbit One or Jawbone UP and ActiGraph outcomes ([Fitbit One or Jawbone UP + ActiGraph outcome]/2) [31]. Cohen’s kappa (κ) statistic was used to assess the agreement between devices for classification of active versus inactive based first on achieving 10,000 steps or more per day (default step goal on both devices) and second on achieving 30 minutes/day or more of MVPA, comparable with public health guidelines [32]. For each outcome (steps and MVPA), each day was coded as either 0 for active (≥10,000 steps or ≥30 mins of MVPA) or 1 for inactive (<10,000 steps or <30 mins of MVPA). The strength of Cohen’s kappa was interpreted based on the following definitions: less than chance agreement (<.0), slight agreement (.01-.20), fair agreement (.21-.40), moderate agreement (.41-.60), substantial agreement (.61-.80), and almost perfect agreement (.81-.99). *P* values were based on two-sided tests and were considered statistically significant at *P*<.05. All statistical analyses were conducted in SPSS version 21 (IBM Corp, Armonk, NY, USA). Post hoc power calculations determined that a sample size of N=289 daily comparisons would detect correlations as low as .17 with 80% power and 5% alpha.

## Results

### Participant Characteristics

A total of 48 participants were recruited for the larger intervention study (n=16 per group). Of the 32 participants allocated to the activity tracker groups, 29 provided valid data for the current analyses, with comparable numbers of participants in the Fitbit One group (n=14) and the Jawbone UP group (n=15). The characteristics of the participants are presented in Table 1. The sample consisted predominantly of middle-aged women (female: 90%, 26/29; age: mean 39.6, SD 11.0 years) who were highly educated (86%, 25/29 completed tertiary education) and with normal-overweight BMI (mean 25.9, SD 5.0 kg/m^2^).

**Table 1:** Participant characteristics.

Characteristics	All (n=29)	Fitbit (n=14)	Jawbone (n=15)
Female, n (%)	26 (90)	12 (86)	14 (93)
Age (years), mean (SD)	39.6 (11.0)	36.1 (12.8)	42.8 (8.1)
Completed tertiary education, n (%)	25 (86)	13 (93)	12 (80)
BMI (kg/m^2^), mean (SD)	25.9 (5.0)	26.5 (6.5)	25.4 (3.2)

### Comparison Findings

The 29 participants contributed a total of 289 valid days of data for analyses (Fitbit One: n=135 days; Jawbone UP: n=154 days). Descriptive statistics, correlation coefficients, agreement, and Bland-Altman parameters for Fitbit One, Jawbone UP, and ActiGraph are presented in Table 2. According to accelerometry estimates, participants in the Fitbit group accumulated a mean 8497 (SD 2878) steps/day and 36.6 (SD 25.0) minutes/day of MVPA. Mean values for the Jawbone UP group were 7511 (SD 2692) steps/day and 37.4 (SD 22.8) minutes/day of MVPA; the mean longest sedentary bout for this group was 46.4 (SD 9.8) minutes/day. Overall, correlations for steps and MVPA were strong for both devices, although higher for Fitbit One (*r*=.85 for steps and ρ=.80 for MVPA) than for Jawbone UP (*r*=.75 for steps and ρ=.75 for MVPA). The correlation between Jawbone UP longest idle time and ActiGraph longest sedentary bout was poor (ρ=.19). Absolute agreement (ICC) was acceptable for ActiGraph and Fitbit One steps (.90) and MVPA (.72) and Jawbone UP steps (.79). However, agreement was weak between ActiGraph and Jawbone UP estimates of MVPA (.56) and longest idle time (.08).

**Table 2:** Descriptive statistics, correlations, agreement, and Bland-Altman parameters for Fitbit One and ActiGraph GT3X+.^a^

Statistic	Fitbit One		Jawbone UP	
	Steps	MVPA	Steps	MVPA	Longest sedentary bout
Mean (SD)	9221 (3416)	17.0 (17.6)	8690 (4029)	75.5 (35.5)	87.1 (45.6)
GT3X+, mean (SD)	8497 (2878)	36.6 (25.0)	7511 (2692)	37.4 (22.8)	46.4 (9.8)
*r* /ρ (95% CI)^b^	.85 (.80, .89)	.80 (.73, .85)	.75 (.67, .81)	.75 (.67, .81)	.19 (.03, .34)
ICC (95% CI)^c^	0.90 (0.86, 0.93)	0.72 (–0.15, 0.90)	0.79 (0.72, 0.84)	0.56 (–0.20, 0.83)	0.08 (–0.12, 0.27)
Mean difference (SD)^d^	0.2*x–916.1 (1820)	–0.4*x–9.2 (–19.2)	0.5*x–2491.3 (699.0)	0.5*x+10.6 (38.1)	1.7*x–72.3 (44.8)
95% Limits of agreement^d^					
Upper	(0.2*x–916.1)+3567.0	0.2*x+0.5	(0.5*x–2491.3)+5290.0	0.8*x+20.2	(1.7*x–72.3)+87.8
Lower	(0.2*x–916.1)–3567.0	–0.8*x–18.8	(0.5*x–2491.3)–5290.0	0.08*x+1.1	(1.7*x–72.3)–87.8

^a^Days analyzed for Fitbit One: n=135; days analyzed for Jawbone UP: n=154.

^b^Correlations for steps were calculated using Pearson correlation coefficient (r). Correlations for MVPA and longest idle time were calculated using Spearman rank correlation coefficient (ρ) due to non-normally distributed data. All correlations were significant at *P*<.05.

^c^All agreements are significant at *P*<.001 except for longest sedentary bout (*P*>.99).

^d^Where the mean difference or limits of agreement were systematically biased, equations are presented, where x=a given value on the x-axis (mean of device and ActiGraph GT3X+ value).

**Figure 2:**
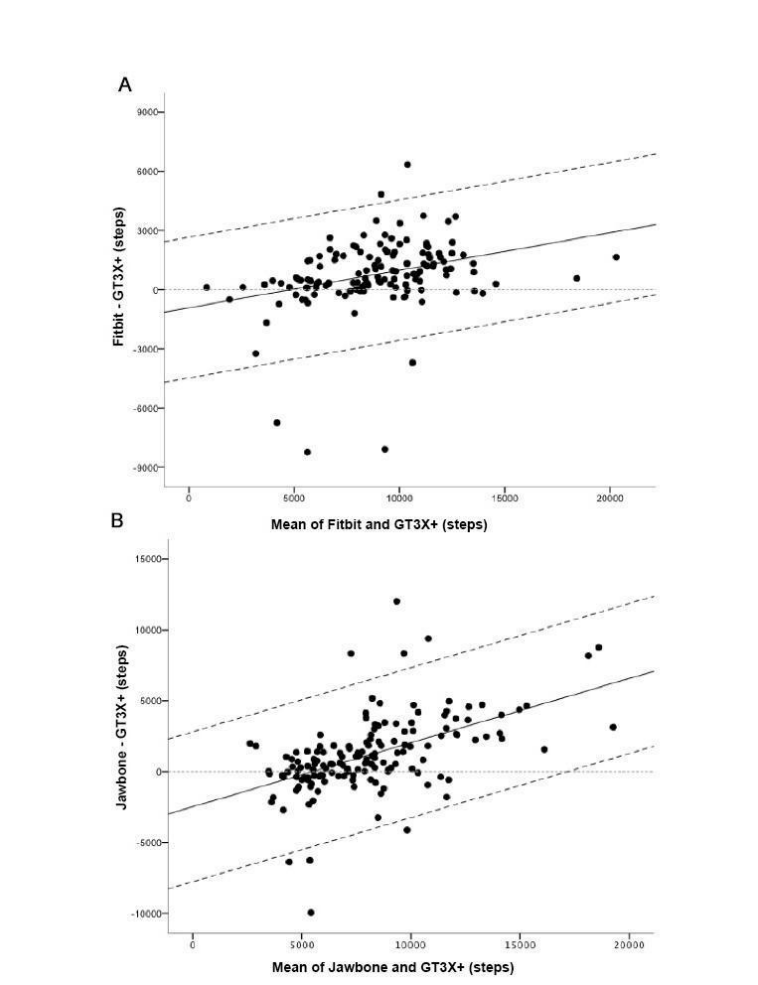
Bland-Altman plots for device steps and ActiGraph steps: Fitbit (panel A; n=135), Jawbone UP (panel B; n=154). The solid line represents the mean difference (steps) between the two measures and the dashed lines are the 95% limits of agreement.

Bland-Altman plots for steps for both devices are presented in Figure 2. For the estimation of steps, analyses revealed systematic bias for mean difference for both devices, with differences increasing with increasing steps/day (see Figure 2). However, 95% limits of agreement were unbiased for both devices and limits were wider for the Jawbone UP than for Fitbit One (5290 and 3567 steps/day, respectively). When absolute values were calculated using the mean of the x-axis values (mean of device and GT3X+ values; Fitbit One: 8859 steps/day; Jawbone UP: 8100 steps/day), both devices overestimated steps (Fitbit One: mean bias 767, 95% limits of agreement –2800 to 4334; Jawbone UP: mean bias 1178, 95% limits of agreement –4112 to 6468).

**Figure 3:**
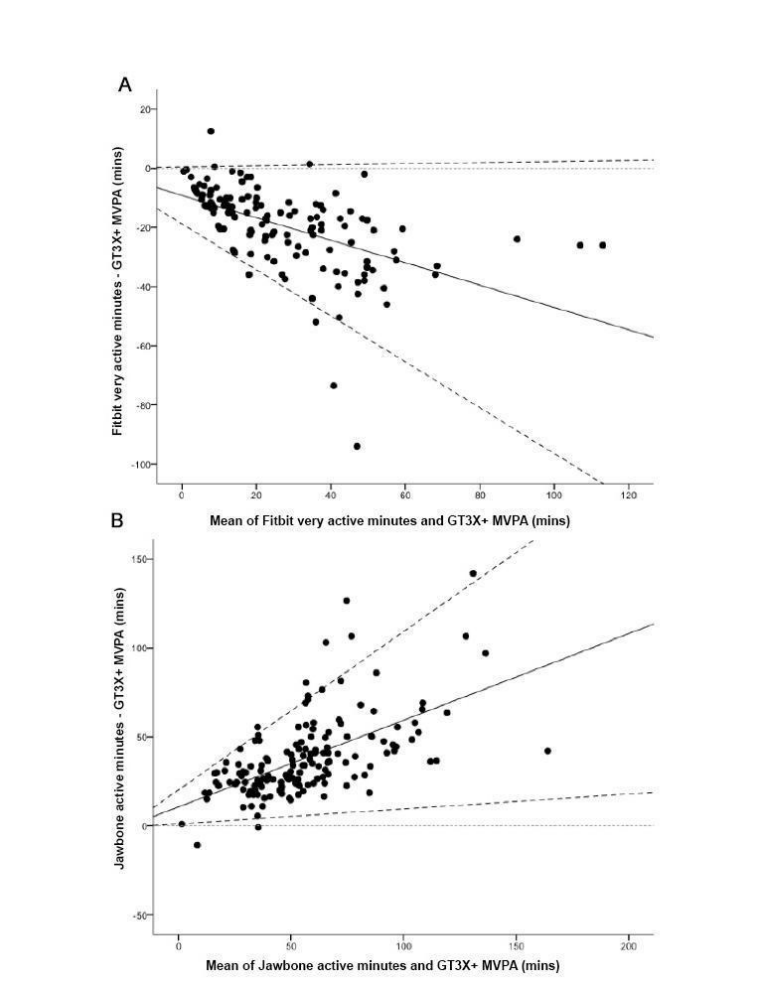
Bland-Altman plots for Fitbit “very active minutes” (panel A; n=135) and Jawbone “active minutes” (panel B; n=154) and ActiGraph MVPA (mins). The solid line represents the mean difference (mins) between the two measures and the dashed lines are the 95% limits of agreement. MVPA: moderate-to-vigorous physical activity.

For MVPA, systematic bias was evident for both the mean difference and the limits of agreement for both the Fitbit One and the Jawbone UP (see Figure 3). The bias was toward larger mean differences and 95% limits of agreement as values on the x-axis increased. When absolute values were calculated using the mean of the x-axis values (mean of device and GT3X+; Fitbit One: 26.6 minutes/day; Jawbone UP: 56.4 minutes/day), the Fitbit One underestimated MVPA by a mean 19.2 minutes/day (95% limits of agreement –39.2 to 5.5), whereas the Jawbone UP overestimated by a mean of 38.1 minutes/day (95% limits of agreement 5.8-65).

**Figure 4:**
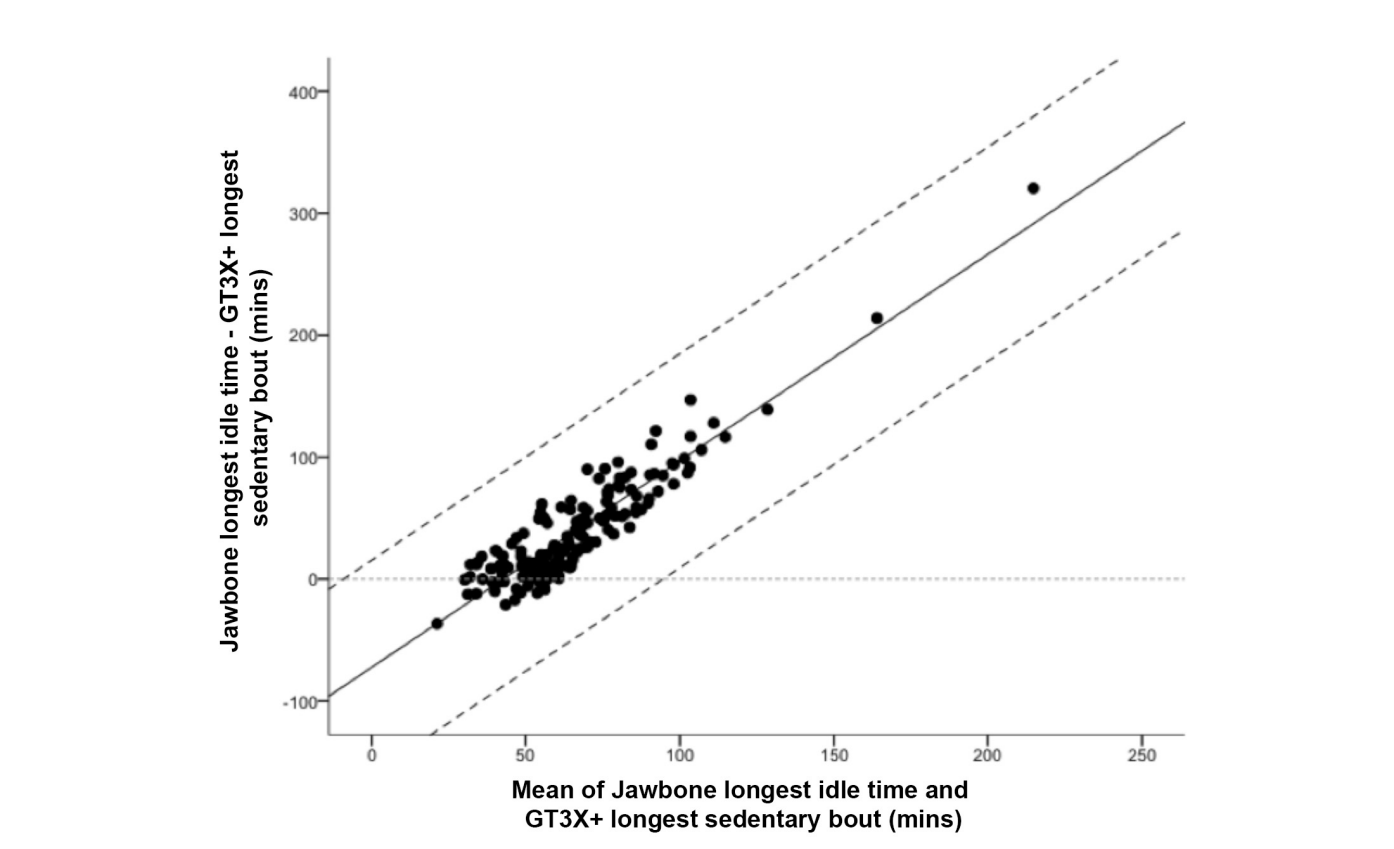
Bland-Altman plot for Jawbone “longest idle time” and ActiGraph “longest sedentary bout.” The solid line represents the mean difference (minutes) between the two measures and the dashed lines are the 95% limits of agreement.

The differences between Jawbone UP and ActiGraph estimates of longest sedentary bout were also biased (see Figure 4), with larger differences when bouts were longer. The limits of agreement were unbiased but wide (mean difference ±88 minutes), varying by up to 150% of the mean estimate according to ActiGraph.

### Classification of Active Versus Inactive

Using the criterion of at least 10,000 steps per day, agreement between the Fitbit One and ActiGraph for the classification of active versus inactive was substantial (κ=.68, *P*<.001). The Fitbit One correctly classified 95% (41/43) of days as active and 79% (73/92) of days as inactive. Agreement between the Jawbone UP and ActiGraph was moderate (κ=.52, *P*<.001). The Jawbone UP correctly classified 90% (25/28) of days as active and 80% (100/126) of days as inactive.

Using the criterion of at least 30 minutes/day of MVPA, agreement between the Fitbit One and ActiGraph was fair (κ=.39, *P*<.001). The Fitbit One correctly classified 40% (28/70) of days as active and 100% (63/63) of days as inactive (<30 min of MVPA per day). Agreement between the Jawbone UP and ActiGraph was slight (κ=.14, *P*=.001). The Jawbone UP correctly classified 100% (94/94) of days as active and 12% (7/60) of days as inactive.

## Discussion

The aim of this study was to compare Fitbit One and Jawbone UP estimates of steps, MVPA, and sedentary behavior to data from the ActiGraph GT3X+ accelerometer in a free-living context. Both the Fitbit One and Jawbone UP demonstrated acceptable accuracy compared with an ActiGraph GT3X+ accelerometer for the estimation of steps per day; however, there were large over- and underestimates of MVPA. Analyses revealed systematic bias for both devices, with significant linear associations between the mean difference and mean values for steps and MVPA, and for the 95% limits of agreement and mean values for MVPA alone. The validity of the Jawbone UP measure of sedentary behavior (“longest idle time”) was poor. Both devices accurately classified more than 80% of the sample days as active or inactive based on the 10,000 steps criterion; however, days were frequently misclassified for meeting public health guidelines of 30 minutes/day of MVPA.

The findings reported in this study suggest that both activity trackers have utility for counting steps in free-living settings, with both devices overestimating daily steps by only 5% to 15% compared with ActiGraph (Fitbit One: 8%; Jawbone UP: 14%). These findings are comparable to those reported in other studies in free-living contexts [16,17], although our correlation for Jawbone UP steps was lower (*r*=.75 vs *r*=.97) [17]. This difference in correlation may be due to the larger sample size in our study (n=154 vs n=21 days). The Fitbit One and Jawbone UP have also been previously assessed in laboratory settings, where the correlation with reference measures was considerably stronger (.97-1.00) [13,15,33]. This is likely due to the tightly controlled conditions in a laboratory protocol. No previous studies have reported systematic bias for steps or MVPA and these findings are important as they suggest that the magnitude and direction of the average device error changes with increasing total number of steps/day. However, this appears to have little influence on the classification of participants as active or inactive based on the cutoff of 10,000 steps/day, with excellent agreement for both devices compared with ActiGraph accelerometry.

Both devices were less accurate measuring MVPA than steps, with correlations of .56 to .80 for both devices against ActiGraph data. These findings are comparable to those reported by Ferguson et al [17], who also reported a similar range of correlations for Fitbit One and Jawbone UP estimates of MVPA compared with ActiGraph accelerometry (.46-.91) in a free-living context. Our findings also demonstrated systematic bias in both devices across mean difference and 95% limits of agreement, indicating that both the difference between devices and the range of error vary across mean values.

It is important to note that despite reasonable correlations for MVPA, compared with ActiGraph, the Fitbit One underestimated MVPA by 46%, misclassifying 60% of days as inactive when they were active, and the Jawbone UP overestimated MVPA by 50%, misclassifying 88% of days as active when they were inactive. The implications of consumer devices over- and underestimating MVPA have significant practical implications. For example, the Jawbone UP overestimated MVPA by a mean 38.1 (SD 22.8) minutes/day and was more likely to classify an inactive day as active. Over a week, this would result in an overestimation of MVPA by a mean of 266.7 (SD 159.6) minutes. Therefore, consumers utilizing these devices will believe that they are engaging in almost twice the recommended dose of physical activity [32], when they are unlikely to be meeting minimum requirements [2].

The large discrepancy in under- and overestimations could be attributable to how MVPA was operationalized. The Fitbit measure of MVPA was the “very active minutes” variable, which is described by the manufacturer as “higher intensity exercise.” The Jawbone measure of MVPA was the “active time” variable, for which no manufacturer’s definition could be identified. We assumed that these measures could relate to MVPA, given that users are encouraged to accrue time spent in these outcomes for health benefits, in line with the promotion of MVPA as “health-enhancing” physical activity. An alternate interpretation of the Fitbit data could be vigorous activity alone, although the correlation was worse for vigorous alone (ρ=.43 compared with ActiGraph) than for MVPA. After data for this study were collected, Fitbit updated this variable with the label of “active minutes” and a clear definition of “any activity that elicits energy expenditure of ≥3 METs [metabolic equivalents] for a duration of 10 minutes or more.” This aligns the Fitbit definition of “active minutes” directly with the US Centers for Disease Control and Prevention recommendations for MVPA [21] and future studies should investigate whether the revised classification improves the accuracy of MVPA estimates. The Jawbone active time data could include activities of intensity less than moderate-vigorous, which could also explain the difference from ActiGraph MVPA data.

The Jawbone UP is one of the few devices available on the market that reports on time spent idle and this is valuable because of the evidence base linking prolonged sedentary behavior to a range of adverse health outcomes [34]. Our findings indicate that the accuracy of these data are poor, with weak correlations between the Jawbone UP variable “longest idle time” per day and ActiGraph-determined “longest sedentary bout” per day. No previous studies have investigated the “idle time” feature of the Jawbone UP and, similar to MVPA, the poor correlations may reflect the relative measures of idle time and sedentary time. Sedentary behavior is now well defined as any waking activity performed in a reclined or seated posture that elicits an energy expenditure of ≤1.5 METs [35]. However, the manufacturer’s definition of “idle time” could not be identified. Our assumption that idle time should relate to sedentary time has good justification because Jawbone UP discourages idle time in the same way that health promotion messages discourage sedentary behavior [32]. Nonetheless, increased transparency from manufacturers regarding exact definitions of their variables and how they are calculated (including both idle time and active time for the Jawbone UP) would significantly improve the ability of researchers to explore the accuracy of these devices. It would also inform decision making about how to use these monitors in research studies.

In a recent systematic review of intervention studies, self-monitoring was reported as a “very promising” tool for reduction of sedentary behavior [36]. Accurate tools for self-monitoring sedentary behavior are critically needed; however, our findings suggest that the Jawbone UP measure of idle time should be used with caution. This is an important finding given that the Jawbone UP measures of idle time and the associated “inactivity alerts” are a point of difference between the many devices that are available.

Limitations of this study include the predominantly female, healthy, middle-aged sample, which limits the generalizability of the findings. The study could not control for wear time of the consumer devices and this may explain in part some of the large absolute differences between the devices and the ActiGraph. Although it is likely that data with very large differences may be attributable to differences in wear time, these data were not excluded from the current analyses because we were not able to verify this objectively. However, the sample had good wear compliance, evidenced by mean daily steps above normative values for this population [3]. In addition, it is possible that comparing devices with two different wear locations (wrist-worn Jawbone UP vs hip-worn ActiGraph) may have influenced the results. However, a recent study has reported that ActiGraph measured physical activity correlates moderately well between wrist and hip sites [37]. Further, this study used the ActiGraph with the cutpoint of 100 counts per minute as the comparison methods for sedentary behavior. Although this approach is commonly used in this field [5,24], future studies should consider using the activPAL (PAL Technologies Ltd, Glasgow, UK) device, a thigh-worn accelerometer/inclinometer that evaluates time spent sedentary based on posture rather than the cutpoint method [38]. Finally, the epoch length of the consumer-based trackers may be different from the ActiGraph. Accelerometry data in this study were processed in 30-second epochs. Additional sensitivity analyses (not shown) using 60-second epochs did not alter the overall findings.

Study strengths include the free-living setting, which improves ecological validity and takes previous laboratory studies into a real-world setting. More specifically, our study was conducted in the context of a physical activity intervention as would typically be used in research activities (eg, for self-monitoring of behavior change). The study also concurrently assessed two of the most popular brands of activity trackers on the market and two popular wear locations, wrist and waist. The reference measure (ActiGraph) was a previously validated device, with a large number of daily observations for comparison. Finally, our thorough evaluation of systematic bias is novel and a strength of this study. Only one recent study has assessed the possibility of systematic bias for these devices and only for the outcome energy expenditure [39]. It also found potential for systematic bias in the mean difference, with less evident bias for the Fitbit Flex than the Jawbone UP [39].

Consumer-based activity trackers are widely used in the general population and research settings and have considerable potential to facilitate positive health behavior change in individuals, through the self-monitoring of physical activity and sedentary behavior. From both a consumer and researcher perspective, it is critical that these devices measure what they claim to measure. Our findings suggest that the Fitbit One and Jawbone UP have utility for measuring steps; however, due to modest accuracy and systematic bias, they are better suited as self-monitoring tools (eg, for the public consumer or in behavior change interventions) rather than for evaluation of research outcomes. The outcomes that relate to health-enhancing MVPA (eg, the Fitbit One’s “very active minutes” or the Jawbone UP’s “active time”) and sedentary behavior (“idle time” on the Jawbone UP) should be used with caution for both consumers and researchers alike. Future research should continue to assess the accuracy of these activity and sedentary behavior outcomes as manufacturers refine the measurement capacity of these devices.

## Abbreviations

BMI: body mass index
ICC: intraclass correlation coefficient
MET: metabolic equivalent
MVPA: moderate-to-vigorous physical activity
